# Palmitic acid alters enhancers/super-enhancers near inflammatory and efferocytosis-associated genes in human monocytes

**DOI:** 10.1016/j.jlr.2025.100774

**Published:** 2025-03-09

**Authors:** Vinay Singh Tanwar, Marpadga A Reddy, Suchismita Dey, Vajir Malek, Linda Lanting, Zhuo Chen, Rituparna Ganguly, Rama Natarajan

**Affiliations:** 1 Department of Diabetes Complications and Metabolism, Arthur Riggs Diabetes & Metabolism Research Institute, Beckman Research Institute of City of Hope, Duarte, CA, USA; 2 Irell and Manella Graduate School of Biological Sciences, Beckman Research Institute of City of Hope, Duarte, CA, USA

**Keywords:** free fatty acids (FFA), genomics, inflammation, dyslipidemias, diabetes, obesity, monocyte, macrophage, epigenetic mechanisms, phagocytosis

## Abstract

Free fatty acids like palmitic acid (PA) are elevated in obesity and diabetes and dysregulate monocyte and macrophage functions, contributing to enhanced inflammation in these cardiometabolic diseases. Epigenetic mechanisms regulating enhancer functions play key roles in inflammatory gene expression, but their role in PA-induced monocyte/macrophage dysfunction is unknown. We found that PA treatment altered the epigenetic landscape of enhancers and super-enhancers (SEs) in human monocytes. Integration with RNA-seq data revealed that PA-induced enhancers/SEs correlated with PA-increased expression of inflammatory and immune response genes, while PA-inhibited enhancers correlated with downregulation of phagocytosis and efferocytosis genes. These genes were similarly regulated in macrophages from mouse models of diabetes and accelerated atherosclerosis, human atherosclerosis, and infectious agents. PA-regulated enhancers/SEs harbored SNPs associated with diabetes, obesity, and body mass index indicating disease relevance. We verified increased chromatin interactions between PA-regulated enhancers/SEs and inflammatory gene promoters and reduced interactions at efferocytosis genes. PA-induced gene expression was reduced by inhibitors of BRD4, and NF-κB. PA treatment inhibited phagocytosis and efferocytosis in human macrophages. Together, our findings demonstrate that PA-induced enhancer dynamics at key monocyte/macrophage enhancers/SEs regulate inflammatory and immune genes and responses. Targeting these PA-regulated epigenetic changes could provide novel therapeutic opportunities for cardiometabolic disorders.

Elevated levels of free fatty acids (FFA) are associated with insulin resistance, dyslipidemia, increased low-density lipoprotein, and inflammatory cytokine levels, all of which play crucial roles in cardiometabolic diseases such as obesity, atherosclerosis, and type 2 diabetes (T2D) (1, 2). Palmitic acid (C16:0) (PA), a long-chain saturated fatty acid (FA), is derived from the diet or endogenous synthesis via de novo lipogenesis in the liver and intracellular lipolysis in adipose tissues (2, 3). Experimental and clinical studies suggest that elevated levels of PA from both exogenous and endogenous sources are adversely associated with cardiometabolic disease risk and mortality (3, 4). Palmitate, primarily taken up by cells through the cluster of differentiation 36 (CD36) membrane transporter or via other mechanisms such as albumin-mediated transfer, promotes insulin resistance, adipose tissue inflammation, hepatic steatosis, endothelial dysfunction, and activation of monocyte/macrophages, factors known to increase cardiometabolic risk (2, 5, 6). In obesity, insulin resistance and T2D, PA-induced activation of monocyte/macrophages enhances the production of inflammatory cytokines like tumor necrosis factor-alpha (TNF-α) and Interleukin (IL)-1β, and dysregulates key macrophage functions implicated in inflammatory cardiometabolic diseases (7, 8, 9).

Macrophages exhibit remarkable plasticity and orchestrate both pro-inflammatory and resolution phases in atherosclerosis (7, 10). Treatment of human monocytes with a mixture of free fatty acids containing PA-induced adhesion to endothelial cells, intracellular reactive oxygen species (ROS) formation, CD11b expression, and Protein kinase C activation. PA also induced the expression of *CXCL10* and other pro-inflammatory mediators via NF-κB activation in human macrophages. Conditioned medium from PA-treated macrophages also promoted lymphocyte migration (11). In obesity, macrophages are increased in adipose tissue, where they form crown-like structures around necrotic adipocytes while inflammatory cytokines released by these adipose tissue macrophages (ATMs) contribute to insulin resistance and metabolic dysfunction (12). PA released by adipose tissue not only induces inflammatory responses in macrophages but also impairs macrophage phagocytosis and efferocytosis, key functions of macrophages in homeostatic mechanisms responsible for the clearance of dead cells and inflammation resolution, further contributing to the pathogenesis of inflammatory cardiometabolic diseases (13, 14). While these studies underscore the proinflammatory and adverse effects of PA in monocytes/macrophages, the specific molecular mechanisms regulating pathologic genes, including the potential involvement of epigenetic mechanisms, remain poorly understood.

Enhancers are cis-regulatory elements in the genome that are usually distal to promoters and contain binding sites for multiple transcription factors (TFs). They can control gene expression patterns by interacting with promoters from long distances during development and disease. Genome-wide analyses have demonstrated that specific chromatin signatures are associated with enhancers, including histone H3 lysine 4 mono-methylation (H3K4me1), H3K4me2, and H3K27 acetylation (H3K27ac), which are also helpful in characterizing the transitions of enhancers from inactive to primed or active states, and from activate to inactive states and in regulating genes under diverse biological responses (15, 16). Furthermore, clusters of enhancers known as Super enhancers (SE)s, exhibit even higher densities of TF binding sites and regulate tissue-specific gene expression (17, 18). Enhancer activity is tightly regulated by TFs, coactivators, co-repressors, and histone modifications, which modulate enhancer function via chromatin remodeling to facilitate enhancer-target promoter loops and regulate tissue-specific gene expression (15, 16). Enhancers often harbor genetic variants associated with human disorders, including cardiometabolic disease, and are thus important regulatory regions (19, 20).

Dynamic changes in key histone modifications and subsequent enhancer reprogramming play important roles in regulating genes associated with the differentiation of monocytes to macrophages as well as their phenotypes and functions in inflammatory cardiometabolic disease. (21, 22, 23, 24). Enhancers have emerged as crucial epigenetic regulators of gene expression in diabetes and its complications (25, 26). However, the regulation of enhancers and SEs associated with pathologic gene expression in PA-stimulated human monocytes and macrophages remains unknown.

Here, we investigated correlations between dysregulated enhancers and SEs, enhancer-promoter interactions and gene expression in human CD14^+^ monocytes treated with PA using a combination of histone H3K27ac ChIP-seq, RNA-seq, and promoter capture Hi-C data (PCHi-C), which examines long-range genomic interactions). We found that PA treatment extensively altered the human monocyte enhancer landscape, with upregulated enhancers at inflammatory genes and downregulated enhancers at phagocytosis/efferocytosis-related genes. PA also increased chromatin loop interactions between enhancers and promoters of inflammatory genes (e.g., Interleukin 1 receptor-associated kinase 2 (*IRAK2*), while it inhibited interactions at genes associated with efferocytosis (e.g., MER proto-oncogene, tyrosine kinase (*MERTK*). Genes induced by PA were similarly dysregulated in macrophages exposed to infectious agents and from mice and humans with cardiometabolic diseases. Moreover, we found the enhancer/SE regulator/co-activator Bromodomain containing protein 4 (BRD4) and proinflammatory TF nuclear factor kappa B (NF-kB) play key roles in PA-induced gene expression. PA treatment functionally inhibited phagocytosis as well as efferocytosis in macrophages. In addition, PA-induced enhancers harbored key genetic variants associated with metabolic disorders, and these variants were associated with changes in nearby genes involved in inflammation. These findings provide new insights into epigenetic mechanisms regulated by PA at enhancers/SEs that regulate genes involved in monocyte/macrophage dysfunction relevant to cardiometabolic disease.

## Materials and Methods

### Study Approval

All animal experiments were performed in accordance with the guidelines of the Institutional Animal Care and Use Committee (IACUC), and an approved IACUC protocol #99001 at City of Hope. Experiments with human monocytes were performed in accordance with the guidelines of the Institutional Review Board (IRB) and approved protocol (IRB # 00123). The studies in this work abide by the Declaration of Helsinki principles.

### Animal studies

Male type 2 diabetes db/db mice (BKS.Cg-m+/+leprdb/J, catalog no. 00642), and nondiabetic db/+ littermates, male *Apoe*^*−/−*^mice (8 weeks) on the C57BL/6 background (B6.129P2-Apoetm1Unc/J, catalog no. 002052) were obtained from Jackson Laboratory (Bar Harbor, Maine). Peritoneal macrophages were isolated from control (db/+) and diabetic mice (db/db) mice as described earlier (27, 28). *Apoe*^*−/−*^ mice were injected with vehicle or 5 consecutive low doses of Streptozotocin (50 mg/kg) to induce diabetes (27). 20 weeks later when the *Apoe*^*−/−*^mice developed accelerated atherosclerotic lesions, mice were euthanized, and bone marrow cells were collected from femurs and differentiated into bone marrow-derived macrophages (BMDMs) with M-CSF1 (20 ng/ml, 7 days).

### Cell culture

Human CD14^+^ monocytes were obtained from commercial sources (All Cells) or isolated using peripheral blood monocytes (PBMC) from the blood of normal healthy donors using negative immunomagnetic selection kits (Stem Cells), and cultured in RPMI medium with 10% FBS, penicillin (100 U/ml), streptomycin (100 μg/ml), 2 mmol/L glutamine, 5.5 mmol/L glucose (29). In some experiments, human CD14^+^ monocytes or PBMC were also differentiated into macrophages using M-CSF1 (50 ng/ml) for up to 1 week and treated as indicated. THP1 monocytic cell line (ATCC, catalog no. TIB-202) was cultured in the same medium as primary CD14^+^ monocytes with 50 μM β-mercaptoethanol. THP1 cells were differentiated into macrophages (THP1-macrophage) using PMA (phorbol 12-myristate 13-acetate; 20 ng/ml, up to 48 h). Palmitic acid (PA) stock solution was prepared using 10% BSA. For all experiments in this study, cells were treated with 200 μM of PA or vehicle BSA (0.2%) for 24 h unless otherwise indicated. In some experiments, cells were pre-treated with the BRD4 inhibitor JQ1 (0.5 μM) for 2 h or the NF-κB inhibitor BAY 11–7082 (5 μM), for 1 h, and then PA solution was added to the wells and incubated for additional 24 h. Jurkat T cell line was obtained from (ATCC, catalog no. TIB-152) and cultured in RPMI medium supplemented with 10% FBS and penicillin/streptomycin.

### RNA isolation and RT-q-PCR

RNA was isolated with RNeasy mini (Qiagen, catalog no. 74106) or Direct-zol RNA isolation kits (Zymo Research, catalog no. R2063) according to the manufacturer’s instructions and reverse transcription-quantitative polymerase chain reaction (RT-qPCR) reactions were performed as described earlier (29, 30). Briefly, up to 1 μg of total RNA was converted to cDNA using Prime Script^TM^RT Master Mix (Cat. No. RR036A, Takara), and cDNA was used for qPCR with gene-specific primers. Primers used in the manuscript are listed in supplemental Table S1.

### ChIP and ChIP-Seq

Human CD14^+^ monocytes were treated with PA (200 μM) or vehicle BSA for 24 h. After treatment, cells were fixed using 1% formaldehyde for 10 min, and glycine (125 mM) was added, followed by incubation for 5 min at room temperature. Then cells were washed twice with PBS containing protease inhibitor (Roche, cat#04693132001). Cells were lysed and sonicated for 10 cycles (30 s on 30 s off cycle) using Bioruptor Pico sonicator (Diagenode). About 10% of chromatin from each reaction was saved as input DNA. An equal amount of chromatin from each sample was incubated overnight at 4°C with an antibody to histone H3K27ac (Abcam, Cat # ab4729) or rabbit IgG (negative control for ChIP). Following incubation, magnetic protein G Dynabeads (25 μl, Invitrogen, catalog no. 10004D) were added to capture the immune complexes and incubated for 2 h at 4°C. Subsequently, beads were washed with buffers containing low salt, high salt, and LiCl as described earlier (27, 31). The H3K27ac enriched DNA samples were isolated by reverse-crosslinking by incubation at 65°C overnight. Then, proteins were digested with proteinase K and DNA was isolated using phenol-chloroform extraction followed by ethanol precipitation. The ChIP-seq libraries were prepared from two biological replicates (N = 2) and high-throughput sequencing was performed at the City of Hope’s Integrative Genomics Core as described earlier (31). ChIP DNA was also analyzed by qPCR using indicated enhancer or promoter primers.

### Phagocytosis assays

Human PBMC-derived macrophages were treated with PA (200 μM) for 24 h. Phagocytosis was performed using the Vybrant phagocytosis assay kit (ThermoFisher, V6694) as described earlier (27). Briefly, 100,000 macrophages were plated in a 96-well plate for 2 h followed by incubation with fluoresceine-labeled *E. coli* bioparticles and 2h later, trypan blue was added to quench fluorescence from extracellular beads. Fluorescence from phagocytosed beads was measured using a 96-well plate reader (Tecan Infinity 200 Pro). Images were collected with a 20X objective using a fluorescence microscope (Keyence).

### Efferocytosis assays

These assays were performed with an Efferocytosis Assay Kit (Cayman Chemical, 601,770) according to the manufacturer’s protocol. THP1-macrophages (effector cells) were treated with PA (200 μM) for 24 h. Then, 100,000 cells were labeled with CytoTell Blue, plated in two chamber slides, and incubated for 2 h. Following incubation, apoptotic Jurkat cells (apoptosis was induced by Staurosporine) labeled with carboxyfluorescein succinimidyl ester (CFSE, green) used as bait cells were added, and incubated for another 2 h. Then, images were acquired with the fluorescence microscope using a 20X objective (Keyence) or confocal microscope (Zeiss LSM700) with 40x/1.2NA with a water immersion objective) to determine the macrophages that have efferocytosed apoptotic Jurkat cells.

### ChIP-seq data analysis, enhancer, and super-enhancer (SE) identification

Reads were aligned on the hg19 human genome version with Novoalign (V3.02.07). H3K27ac peaks were called based on MACS2 (version2.1.1.20160309, q-value 0.05). Peaks present in both replicates were selected using the csaw package in R and included in the analysis. H3K27ac peaks present in promoters of coding genes and ENCODE blacklisted regions were excluded. Differential analyses of enhancers were carried out using the edgeR package in R software. Super enhancer analysis was performed using the ROSE algorithm (17). Differentially regulated enhancers were selected based on log2fold change ±1 and a q-value of < 0.05. Differentially regulated super-enhancers were identified depending on log 2-fold changes ±0.8 and q-value of <0.05. Density plot and motif enrichment analyses (findMotifs.pl) were performed using HOMER software. Gene ontology biological process analysis of enhancers was performed using GREAT tools (32).

### SNP analysis in PA-regulated enhancers

The SNPs associated with T2D, BMI, and obesity (7830) were obtained from published GWAS studies (33). Total PA-regulated enhancer regions (FDR<0.05, n = 861) and non-PA-regulated enhancers (FDR>0.05, N = 9,370) overlapping with these SNPs were identified using intersectBed command in bedtools (34). A two-sided Fisher’s exact test was used to determine the statistical significance of the enrichment of these GWAS SNPs in PA-regulated enhancer regions.

### Chromatin conformation capture (3C) assays

These assays were performed using published protocols (35). THP1-macrophages treated with 200 μM PA or BSA (24 h) were removed from the plates using accutase (Thermo Fisher Scientific), counted, and fixed using 1% formaldehyde for 10 min and glycine 150 mM was added to the cells and incubated for another 5 min at room temperature. After two washes with PBS containing a protease inhibitor, cells were lysed using cell lysis buffer (15 mM Tris-HCL pH 8.0, 10 mM EDTA,1% SDS, and protease inhibitor) for 10 min on ice followed by centrifugation. The nuclei pellet was resuspended in a cut smart buffer (New England Biolabs) and incubated with Triton X-100 (2%) to quench the SDS at 37°C for 1 Hour. Then, HindIII restriction enzyme was added (3000 U/nuclei pellet from 1.5 million cells) and kept on thermo mixture with shaking at 37°C overnight. The next day samples were diluted up to 2 ml with ligase buffer (New England Biolabs) and incubated overnight at 16°C with shaking. After incubation, samples were extracted using phenol:chloroform, isoamyl alcohol mixture, DNA from the aqueous layer was precipitated with ethanol and DNA pellets dissolved in 30–50 μl of 10 mM Tris pH 7.5. The 3 C DNA template was tested for quality using primers amplifying ALDOA (Aldolase, amplifying the non-HindIII RE sites) and AHF64 (positive control primers amplifying the HindIII RE sites). Once confirmed, qPCRs were performed with 3C DNA from PA- and BSA-treated cells using primers specific for indicated candidate enhancers-promoter pairs. The Ct values were normalized using ALDO-A primers amplifying the non-HindIII restriction enzyme sites, and results were expressed as fold-over BSA-treated samples. The PCHi-C data was downloaded from Blueprint Epigenome (36).

### Statistics

Data are presented as mean ± SD of the experiments performed at least in triplicate. Shapiro-Wilk normality test was used to test the normal distribution of each sample group. Statistical significance was calculated using unpaired 2-tailed Student t-tests (for 2 samples) or 1-way ANOVA with post-tests (for >2 groups) using GraphPad Prism software (8.0 or above) and *P* < 0.05 was considered significant. Bar plots were created using GraphPad Prism software.

## Results

### PA treatment leads to dynamic alterations in the H3K27ac enhancer mark in human monocytes

**Fig. 1:**
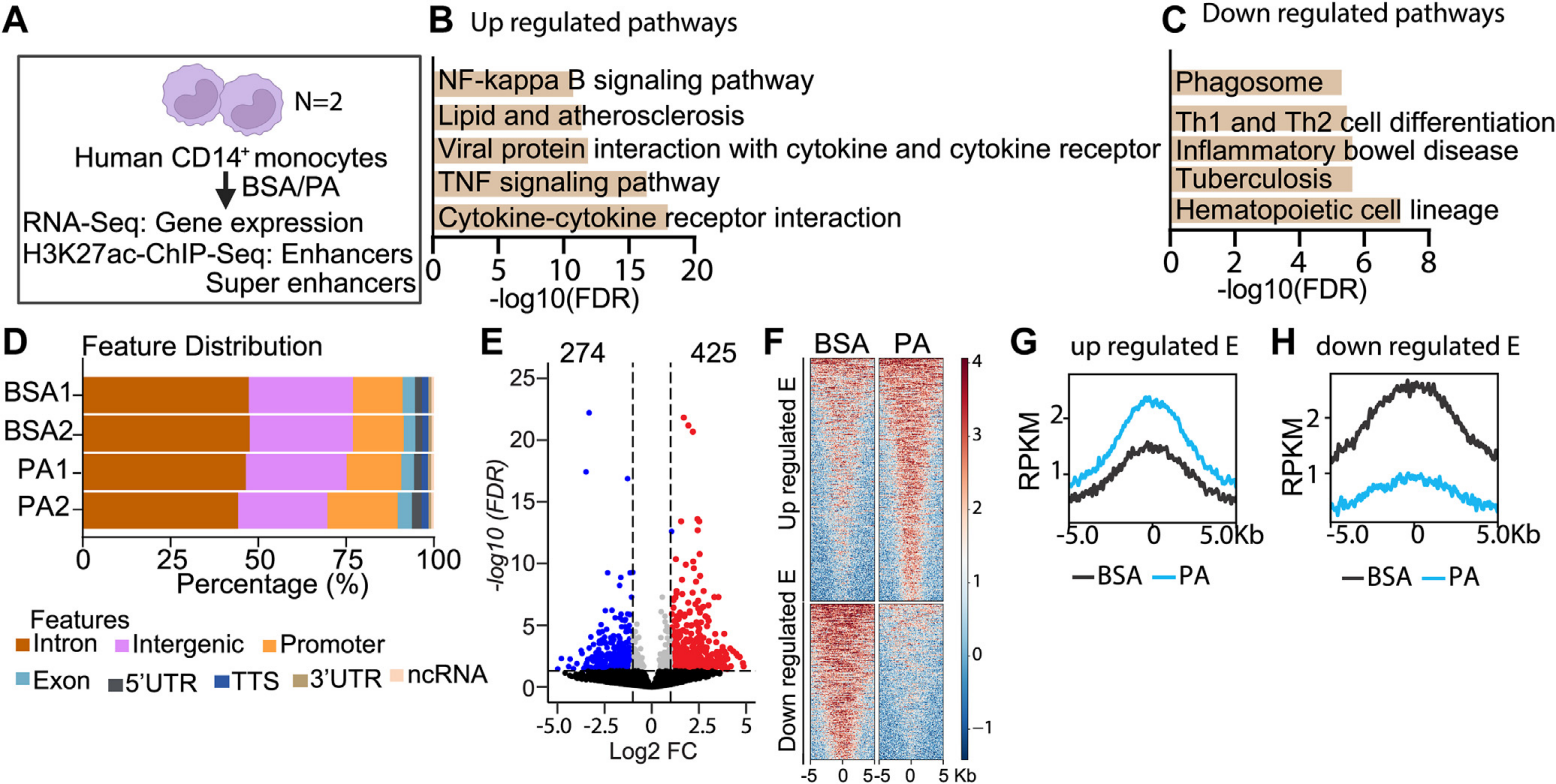
PA-induced gene expression is associated with genome-wide alterations in enhancer repertoire in human CD14^+^ monocytes. A: Experimental design used to identify PA-induced enhancers and super-enhancers in human CD14^+^ monocytes. RNA-sequencing and ChIP-seq were performed on two biological replicates in each condition. B and C: Bar graph showing pathways regulated by the upregulated (B) and downregulated (C) genes in human CD14^+^ monocytes treated with PA. Pathway analysis was performed using DAVID online tools. False discovery rate (FDR)-corrected *P*-values <0.05 were considered significant. D: Genomic distribution of H3K27ac peaks in control vehicle (BSA) and PA treated human CD14^+^ monocytes. E: Volcano plot of the differentially enriched H3K27ac peaks in PA treated human CD14^+^ monocytes versus control (BSA) (log2 fold change [log2FC] ≥1 and FDR ≤0.05). F: Heatmaps of read densities around the enhancer peaks (±5kb from the centre of the enhancer peaks). Red color represents increased and blue color represents decreased read densities. G, H: Graphs showing the read densities of H3K27ac enrichment around up and down regulated enhancers. Black color represents BSA and blue color PA treated human CD14^+^ monocytes.

Using RNA-seq analysis, we recently reported dysregulated expression of key genes and long noncoding RNAs (lncRNAs) in human CD14^+^ monocytes treated with physiologically relevant concentrations of PA (200 μM, 24 h) (30). To determine the previously un-investigated role of enhancers and SEs mediating PA-induced gene expression and monocyte dysfunction, we treated CD14^+^ monocytes isolated from the blood of healthy human volunteers with PA (200 μM) for 24 h or vehicle BSA (0.2%) as control. We performed chromatin immunoprecipitation followed by sequencing (ChIP-seq) with antibodies against H3K27ac (chromatin mark of active enhancers/SEs), and integrated this ChIP-seq data with our RNA-seq datasets (30) to determine the correlation between enhancer activation and gene expression (Fig. 1A). Analysis of RNA-seq data using DAVID pathway enrichment software showed enrichment of several inflammatory pathways in PA-treated cells versus control cells, including cytokine-cytokine receptor interactions, Tumor necrosis factor (TNF) signaling, lipid and atherosclerosis signaling, NF-κB signaling and viral infection among the upregulated genes (Fig. 1B). On the other hand, downregulated genes showed enrichment of the hematopoietic cell lineage, and the phagosome pathway (Fig. 1C).

Since PA can be taken up in cells via CD36 (fatty acid transporter), we examined the effect of PA on *CD36* expression. We treated human THP1-macrophages and CD14^+^ macrophages with BSA or PA (200 μM) for 6 h or 24 h followed by gene expression analysis. Results showed that PA treatment increased the expression of *CD36* (supplemental Fig. S1A, B).

These results confirmed that PA regulates key monocyte/macrophage signaling and biological processes/functions associated with inflammation, immunity, defense against pathogens, and pathogenesis of cardiometabolic disease.

ChIP-seq data analyses showed that the H3K27ac mark was enriched at various genomic locations (28-35k peaks in each replicate) across the human monocyte genome in both BSA and PA-treated samples, with a majority of the peaks enriched at introns, intergenic regions, promoters and exons (Fig. 1D). Since H3K27ac marks both active promoters and enhancers, to specifically identify enhancers, we excluded promoter regions of the RefSeq genes and performed a differential analysis of H3K27ac enrichment between PA versus vehicle (BSA) -treated cells. Results showed increased enrichment of H3K27ac (upregulated) at 425 enhancers and reduced H3K27ac (downregulated) at 274 enhancers [log2 fold change (log2FC) ≥1 and false discovery rate (FDR) ≤0.05] in PA-treated CD14^+^ human monocytes versus BSA controls (Fig. 1E). We also plotted the heat map of H3K27ac read densities around ±5 kb from the peak summit for up and down-regulated enhancers, which showed clear differences in H3K27ac enrichment in PA versus BSA (Fig. 1F). Furthermore, profiles of H3K27ac peak densities also confirmed enrichment of H3K27ac at PA-upregulated enhancers and reduced H3K27ac at PA-downregulated enhancers (Fig. 1G, H, respectively). These results demonstrate that PA treatment significantly alters gene expression related to key immune and inflammatory processes with corresponding dynamic changes in enhancers in human CD14^+^ monocytes.

### Alteration in enhancers associated with inflammatory and efferocytosis-related genes

**Fig. 2:**
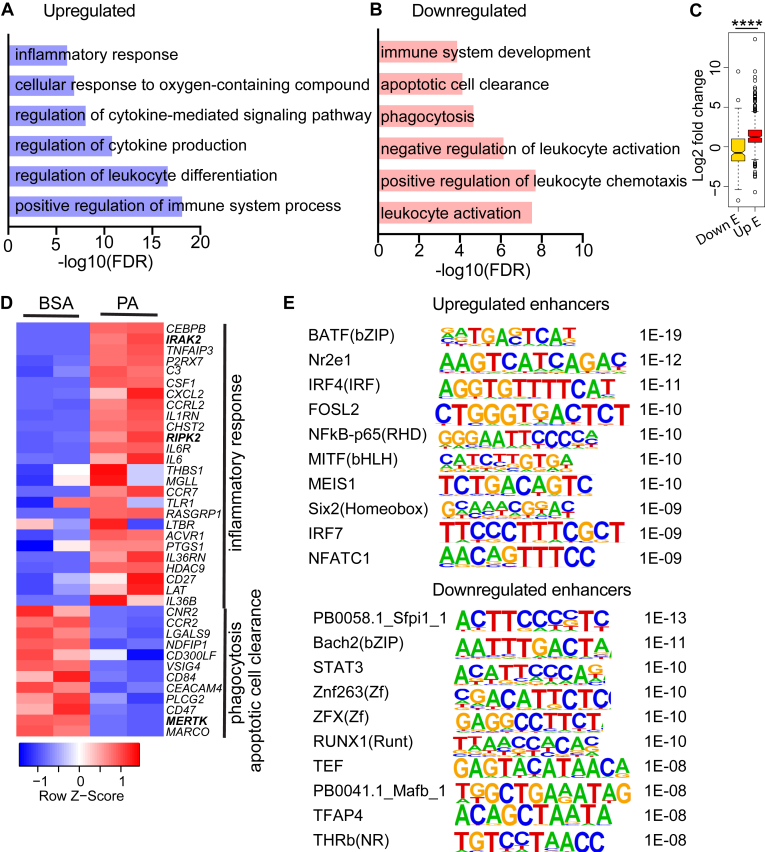
Enhancers regulated by PA are associated with increased expression of inflammatory genes, and reduced expression of genes associated with phagocytosis and efferocytosis. A, B: Bar graphs showing the enrichment of biological processes (BPs) in PA-upregulated (A) and PA-downregulated (B) enhancers. Differentially expressed enhancers were functionally annotated using Genomic Regions Enrichment of Annotations Tool (GREAT). Enhancer-associated genes were selected based on GREAT analysis (two nearest genes in ±1000kb from enhancer location). C: Box plot showing the relation between downregulated enhancers (Down E) and Upregulated enhancers (Up E) and associated gene expression. Expression values (log2 fold change) of the enhancer associated genes (±1000kb) were obtained from RNA-seq datasets of CD14^+^ monocytes treated with BSA and PA. Box plot shows the median and upper and lower quartile range. ∗∗∗∗*P* < 0.0001 as determined by unpaired t-tests. D: Heatmap of genes (shown on right) associated with enhancers in BSA and PA treated cells that have functions related to inflammatory response, phagocytosis, and apoptotic cell clearance. The genes were selected from biological processes (BPs) enriched from GREAT analysis (Inflammatory response, phagocytosis, and apoptotic cell clearance) shown in panels A, B. The heatmap was generated using RPKM values from RNA-seq data sets of CD14^+^ monocytes treated with BSA and PA. E: Enriched transcription factor motifs in upregulated and downregulated enhancers in PA-treated cells.

We performed GREAT analysis to determine the Gene Ontology biological processes (GO-BP) enriched among genes associated with the differentially regulated enhancers in PA-treated monocytes. Results showed that up-regulated enhancers were enriched with positive regulation of immune system processes, leukocyte differentiation, cytokine production and signaling, and inflammatory responses (Fig. 2A). Downregulated enhancers were enriched with leukocyte activation, chemotaxis, immune system development, apoptotic cell clearance, and phagocytosis (Fig. 2B). Since upregulated enrichment of H3K27ac indicates activated enhancers and vice versa, we integrated H3K27ac data with RNA-seq data to examine correlations with the expression of nearby genes (±500 kb) after PA treatment. Results showed a significant correlation between upregulated enhancers and increased expression of nearby genes, while downregulated enhancers were associated with decreased gene expression (Fig. 2C). Interestingly, H3K27ac was increased at enhancers near several upregulated inflammatory response-related genes including *IRAK2*, receptor-interacting serine/threonine kinase 2 (*RIPK2*), Interleukin 6 (*IL6*), Thrombospondin 1 (*THBS1*), and Toll-like receptor 1 (*TLR1*) (Fig. 2D). In contrast, H3K27ac was downregulated at enhancers near phagocytosis and apoptotic cell clearance genes such as *MERTK*, C-C motif chemokine receptor 2 (*CCR2*), and Galectin 9 (*LGALS9*), which were significantly downregulated (Fig. 2D), suggesting enhancers inhibited by PA are involved in regulating genes with functions related to phagocytosis and efferocytosis.

Next, to understand the transcription regulators of the enhancers, we performed a transcription factor (TF) motif enrichment analysis of the upregulated enhancers. Binding sites for inflammation-related TFs like NFκB-p65, Interferon Regulatory Factor 4 (IRF4), IRF7 and Nuclear factor of activated T cells 1 (NFATC1) were enriched among upregulated enhancers, suggesting these TFs regulate functions of enhancers associated with inflammatory processes in PA-treated human CD14^+^ monocytes (Fig. 2E). On the other hand, downregulated enhancers showed enrichment of TFs like Sfpi1 (PU.1), Signal transducer and activator of transcription 3 (STAT3), BTB domain and CNC homolog 2 (BACH2), RUNX family transcription factor 1 (RUNX1), and Transcription factor AP-4 (TFAP4) (Fig. 2E), which are known to modulate macrophage functions and alternative activation.

### Enhancers dysregulated by PA overlap obesity and diabetes-associated single-nucleotide polymorphisms (SNPs) identified from genome-wide association studies (GWAS)

**Fig. 3:**
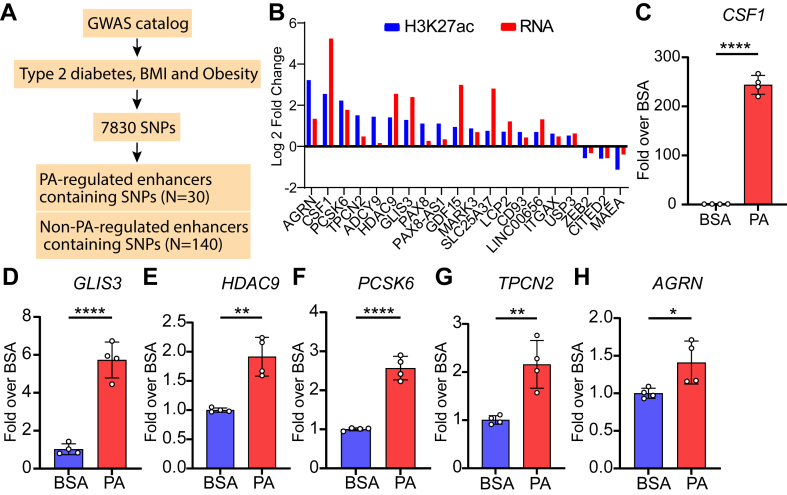
PA-regulated enhancers harbor single-nucleotide polymorphisms (SNP)s associated with metabolic diseases. A: Workflow for identification of SNPs associated with type 2 diabetes, body mass index (BMI), and obesity that are located in PA-regulated enhancers and non-PA-regulated enhancers. B: The effect of PA-regulated enhancers(H3K27ac) overlapping with metabolic disease-associated SNPs on related gene expression (RNA). Data represents log2Fold changes in H3K27ac enrichment (ChIP-seq) and gene expression (RNA-seq) in CD14^+^ monocytes treated with PA versus BSA. C–H: RT-qPCR validation of PA-induced genes associated with enhancers harboring metabolic disease SNPs. THP1 monocytes were differentiated into macrophages and treated with BSA or PA for 24 h followed by RT-qPCR analysis of indicated genes. Error bars represent mean ± SD. ∗*P* < 0.05, ∗∗*P* < 0.01 and ∗∗∗∗*P* < 0.0001, as determined by unpaired t-tests in C–H (n = 4).

GWAS efforts have mapped SNPs in thousands of loci associated with complex metabolic disorders. The majority of these genetic variations are enriched in the non-coding regions of the genome (like enhancers), suggesting key roles for these genetic variations on the functions of these chromatin regulatory elements (20). To examine the statistical enrichment of genetic variants associated with obesity and diabetes in PA-regulated enhancers, 7830 SNPs associated with 3 traits (T2D, obesity, and BMI) were retrieved from published GWAS studies (33) (Fig. 3A). Then we analyzed their overlaps with PA-regulated enhancers (FDR <0.05, N = 861) and non-PA-regulated enhancers (N = 9,370) and identified 30 PA-regulated and 140 non-PA-regulated enhancers containing at least one of these SNPs (Fig. 3A, and supplemental Tables S2 and S3). Two-sided Fisher's exact test showed SNPs associated with the 3 traits that are enriched in PA-regulated enhancer regions compared to the non-PA-regulated enhancers (odds ratio = 2.38, *P*-value = 0.0001).

Furthermore, we examined whether these PA-regulated enhancers harboring SNPs also showed changes in the expression of nearby genes in our RNA-seq data set (PA vs. BSA). We found that several nearby genes, including Colony stimulating factor 1 (*CSF1*), GLIS family zinc finger 3 (*GLIS3*), Histone deacetylase 9 (*HDAC9*), Proprotein convertase subtilisin/kexin type 6 *(PCSK6*), Two pore segment channel 2 (*TPCN2*), and Agrin (*AGRN*) showed increases in expression as well as H3K27ac levels at enhancers (Fig. 3B). Conversely, reduced H3K27ac enrichment at the downregulated enhancers with SNPs correlated with reduced expression of nearby genes (Fig. 3B) such as Macrophage erythroblast attacher, E3 ubiquitin ligase *(MAEA*) gene related to autophagy (37), and transcription repressors Zinc finger E-box-binding homeobox 2 (*ZEB2*) which regulates monocyte-macrophage functions (38) as well as Cbp/P300 Interacting Transactivator With Glu/Asp Rich (*CITED2*), a negative regulator of macrophage pro-inflammatory phenotype (39). Furthermore, we validated the changes in the expression of genes near PA-regulated enhancers harboring SNPs in human THP1-macrophages treated with PA (200 μM) or BSA for 24 h. Results showed that *CSF1, GLIS3*, *HDAC9*, *PCSK6*, *TPCN2*, and *AGRN* were all upregulated by PA versus BSA controls (Fig. 3, C–H), confirming our RNA-seq results. Together, these data suggest that PA-regulated enhancers harboring SNPs might play key roles in the regulation of nearby genes involved in the monocyte/macrophage functions related to BMI, obesity, and diabetes.

### PA-altered super-enhancers (SEs) are enriched in immune, efferocytosis, and phagocytosis-associated genes

**Fig. 4:**
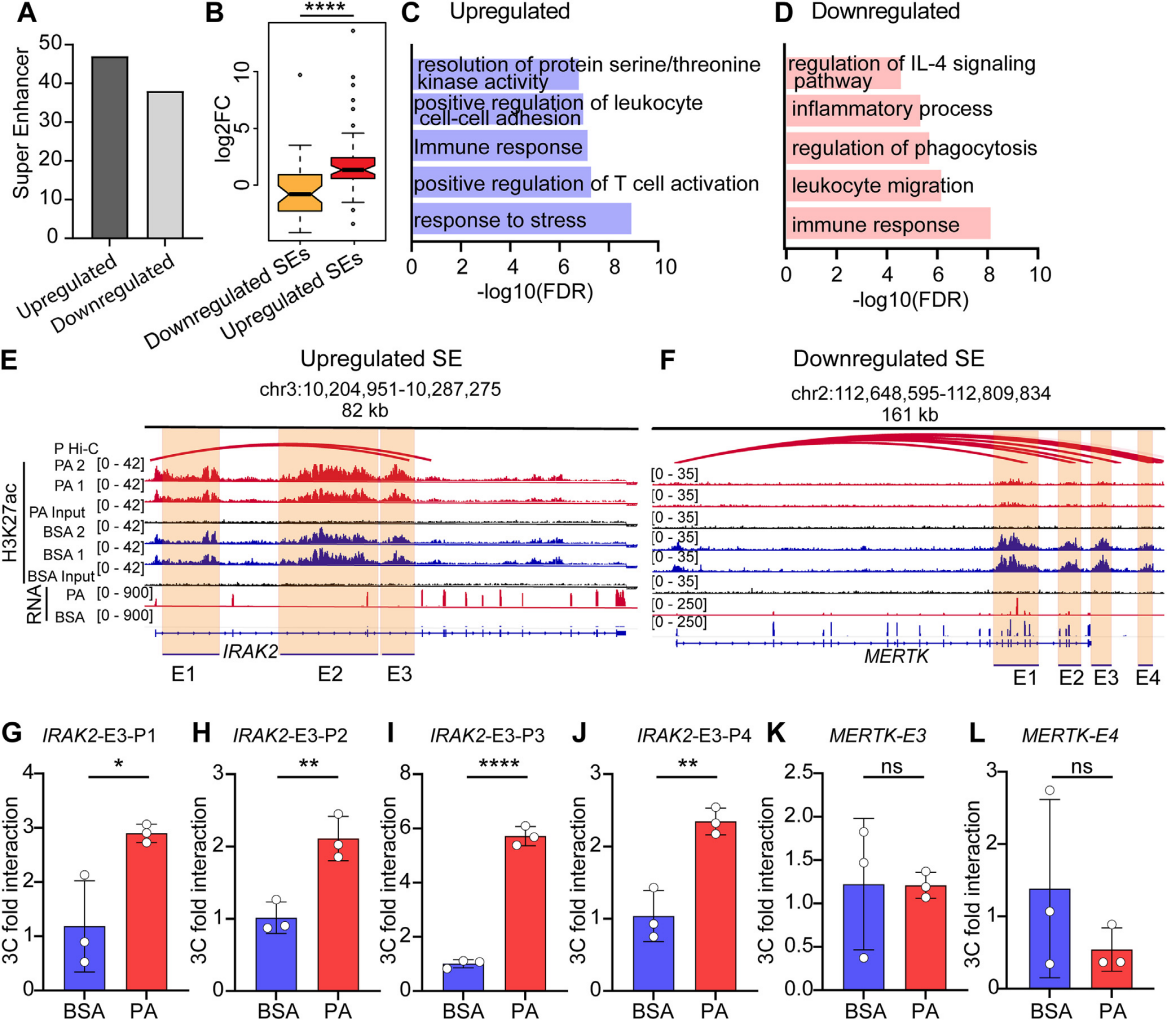
PA-regulated super-enhancers (SE)s are enriched in phagocytosis and other key monocyte phenotypes. A: Graph showing the differentially enriched SEs in PA-treated human CD14^+^ monocytes. B: Box plot showing the association of PA-regulated SEs and gene expression. SEs-associated genes were selected based on GREAT analysis (two nearest genes in ±1000kb from SEs). Box plot shows the median and upper and lower quartile range (n = 2). C, D: Gene ontology biological process analysis of upregulated (C) and downregulated (D) super-enhancers associated genes. E, F: Integration of ChIP-seq (H3K27ac), RNA-seq and publicly available promoter capture Hi-C data shows the upregulated SE around *IRAK2* (E), downregulated SE around *MERTK* gene (F); Vertical orange boxes (E1-E4)- H3K27ac enrichment peaks; Red color loops: chromatin interactions. G–L: qPCR results of the chromosome conformation capture (3C) analysis of enhancer-promoter interactions between SEs and indicated gene promoters in THP1-macrophages treated with BSA or PA for 24 h. (G–L). P1, P2, P3 and P4 represent different primer sets. Error bars represent mean ± SD. ∗*P* < 0.05, ∗∗*P* < 0.01 and ∗∗∗∗*P* < 0.0001, as determined by unpaired t-tests in G–L (n = 3). E1-E4, refer to enhancers shown in panels E, F.

SEs, defined as clusters of enhancers in >12 kb region, regulate cell-specific genes and play important roles in pathophysiological conditions (17). We used the Rose algorithm to identify SEs in our ChIP-seq data and examined whether PA treatment affects the levels and function of key SEs in human CD14^+^ monocytes (17). We found that 47 SEs were upregulated and 38 were downregulated (Log 2-fold change ± 0.8 and FDR≤0.05) (Fig. 4A). Correlations between SEs and the expression of nearby genes revealed that upregulated SEs were associated with highly induced genes and downregulated SEs with reduced gene expression in PA-treated CD14^+^ monocytes (Fig. 4B). Moreover, GREAT analysis showed that upregulated SEs were enriched in GO-BP terms such as response to stress, T cell activation, immune response, cell-cell adhesion, and regulation of serine/threonine kinase activity (Fig. 4C). Whereas downregulated SEs were associated with immune response, leukocyte migration, regulation of phagocytosis, inflammation, and IL-4 signaling pathways (Fig. 4D). These results suggest that SEs regulating immune responses are activated, and SEs regulating phagocytosis-related processes are downregulated by PA in human CD14^+^ monocytes. Next, to determine if differential SEs also interact with promoter regions of nearby genes, we analyzed publicly available PCHi-C datasets (BLUEPRINT) from human CD14^+^ monocytes (36). We found that upregulated SEs around the *IRAK2* genomic region interact with the *IRAK2* promoter regions (Fig. 4E), which could lead to higher *IRAK2* expression in PA-treated cells. In contrast, a downregulated SE at the *MERTK* genomic locus interacted with the *MERTK* promoter regions, suggesting connections between its downregulation and lower *MERTK* expression after PA treatment (Fig. 4F).

Moreover, we performed chromosome conformation capture (3C) assays to experimentally validate the interactions between these enhancers and promoters in human macrophages. We differentiated human THP1-monocytes into macrophages in vitro, treated them with PA (24 h) or vehicle, and performed 3C assays. Results showed that the interactions between enhancers of *IRAK2* with promoter regions increased after treatment with PA (Fig. 4G–J), while 3C assays of downregulated SEs showed a trend towards reduced interactions with nearby *MERTK* promoter (Fig. 4K, L). These results suggest that PA-induced alterations in *IRAK2* and *MERTK* expression are likely regulated via corresponding changes in H3K27ac enrichment as well as promoter-SE interactions in human macrophages. These results support key roles for PA-induced enhancer reprogramming in the regulation of inflammation and efferocytosis functions of human macrophages.

### Treatment with inhibitors of BRD4 and NF-κB attenuates PA-regulated gene expression in macrophages

**Fig. 5:**
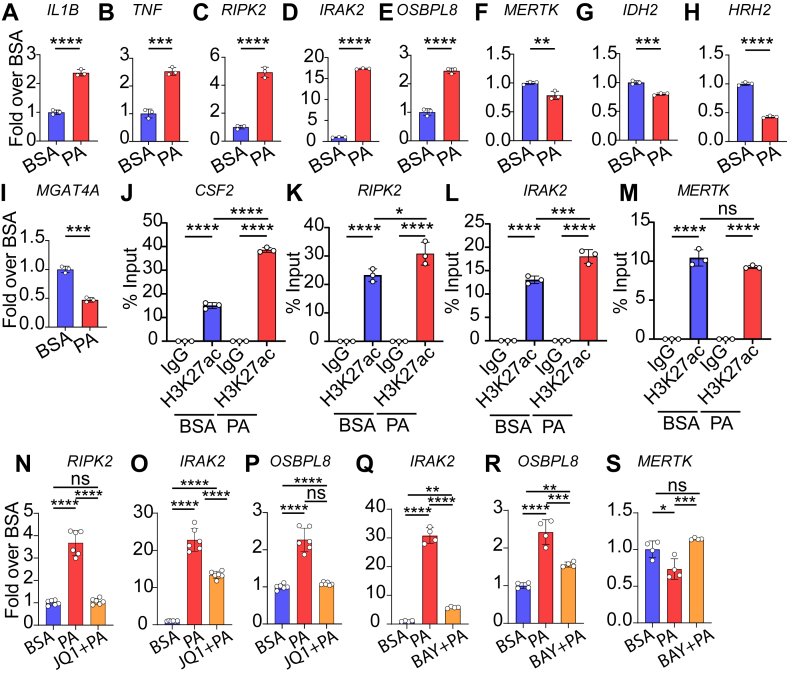
Effects of PA on gene expression in THP-1 macrophages and the effects of inhibitors of BRD4 or NF-kB. A–I: Bar graphs showing the RT-qPCR validation of indicated up-regulated genes (A–E) and downregulated genes (F–I) in THP1-macrophages (TMC) treated with PA (200 μM, 24 h) versus control (BSA) treated TMC. J–M: Validation of PA-regulated SEs by H3K27ac ChIP-qPCR in TMC. TMC were treated with BSA or PA (200 μM) for 24 h. ChIP-assays were performed using H3K27ac specific antibody or IgG control. ChIP-DNA were used for qPCR analysis using indicated SE specific primers for indicated genes. N–P: Gene expression analysis of indicated genes in THP1 macrophages treated with BRD4 inhibitor (JQ1) for 2 h followed by 24 h of PA. Q–S: Gene expression analysis of indicated genes in THP1 macrophages pretreated with NF-κB inhibitor BAY 11–7082 (BAY) for 1 h followed by PA treatment for 24 h. Error bars represent mean ± SD. ∗*P* < 0.05, ∗∗*P* < 0.01, ∗∗∗*P* < 0.001 and ∗∗∗∗*P* < 0.0001 as determined by unpaired t-tests A-I (n = 3) or Ordinary one-way ANOVA with Sidak's multiple comparison test for J–S (J–M, n = 3, N-S, n = 6).

Gene expression analysis using RT-qPCR showed that PA upregulated *IL1B*, *TNF*, *RIPK2*, *IRAK2* and oxysterol binding protein-like 8 (*OSBPL8*) (Fig. 5, A–E), while *MERTK*, isocitrate dehydrogenase (NADP(+)) 2) (*IDH2*), histamine receptor H2 (*HRH2*), and alpha-1,3-mannosyl-glycoprotein 4-beta-N-acetylglucosaminyltransferase A (*MGAT4A*) were downregulated (Fig. 5F–I) in THP1 macrophages showing PA increased the inflammatory genes and decreased efferocytosis associated genes, similar to that seen in CD14^+^ monocytes. Moreover, examination of H2K27ac levels at candidate SEs using ChIP-qPCR showed that H3K27ac enrichment levels were higher at *CSF2*, *RIPK2*, and *IRAK2* SEs (Fig. 5J–L) and decreased at *MERTK* SEs (Fig. 5M) further validating the results observed in CD14^+^ human monocytes.

Next, we evaluated PA-induced gene expression in human macrophages treated with JQ1, an inhibitor of BRD4 and SE functions (40), or BAY 11–7082, an inhibitor of the pro-inflammatory TF NF-κB. THP1 macrophages were pretreated with JQ1 (0.5 μM for 2 h) or BAY 11–7082 (5 μM, 1 h) followed by treatment with PA for 24 h. JQ1 attenuated the expression of *RIPK2*, *IRAK2*, and *OSBPL8*, genes associated with upregulated SEs (Fig. 5N–P). Similarly, treatment with BAY 11–708 attenuated the increased expression of these SE-associated inflammatory genes *IRAK2*, and *OSBPL8* (Fig. 5Q, R), while *MERTK*, which was inhibited by PA, was restored to levels similar to BSA-treated cells (Fig. 5S). These results demonstrate the key roles played by SEs and NF-κB in the PA-induced pathological phenotype of human monocytes and macrophages.

### PA inhibits phagocytosis and efferocytosis in human macrophages

**Fig. 6:**
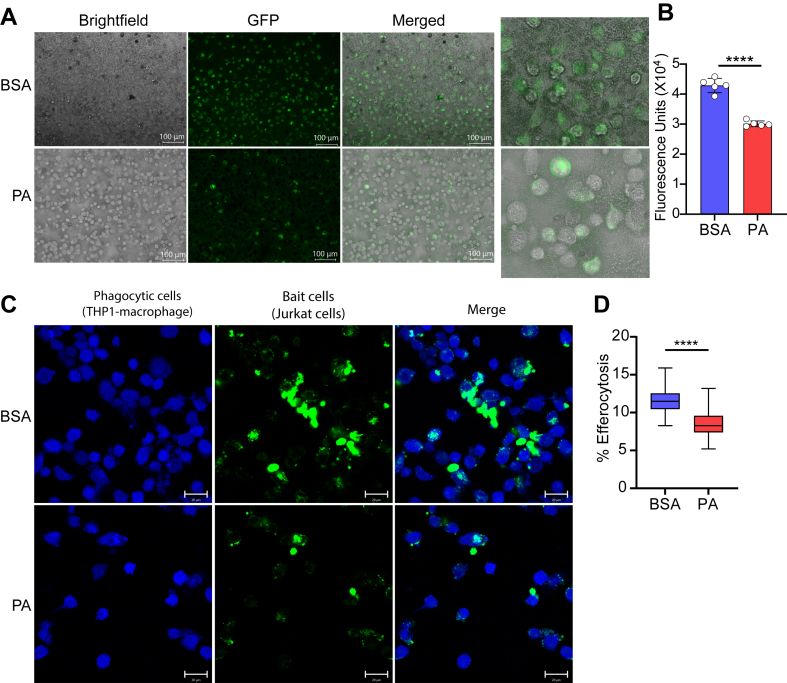
PA inhibits phagocytosis and efferocytosis in human macrophages. A, B: Effect of PA treatment on phagocytosis in macrophages. Representative images showing phagocytosis of *E. coli* bioparticles (green) (A) and quantification of fluorescence intensity (B). THP1-macrophages were treated with BSA or PA for 24 h followed by incubation with fluorescently labeled *E. coli* bioparticles (green). Fluorescence intensity was determined using a 96-well plate reader. Error bars represent mean ± SD. Images were acquired with the fluorescence microscope using a 20X objective (Keyence). C, D: Representative images showing inhibition of efferocytosis in THP1-macrophages treated with PA (200 μM, 24h) versus BSA treated cells. THP1-macrophages treated with BSA or PA (24 h) were incubated with fluorescently labeled apoptotic Jurkat cells (green). Images were taken using a confocal microscope (Zeiss LSM700) with 40x/1.2NA with water immersion objective from n = 4 replicates (C) and quantified using CellProfiler software (D). Box plot on the right shows quantification of efferocytosis expressed as percent of efferocytosis (Cells with efferocytosis X 100/total cells) (D). ∗∗∗∗*P* < 0.0001 as determined by unpaired t-tests for B and D (n = 4–5 images).

Since we observed PA also decreased the expression of efferocytosis/phagosome-related genes in human monocytes/macrophages, next, we examined the direct effects of PA on phagocytosis and efferocytosis in human macrophages. We differentiated human PBMCs into macrophages with M-CSF (50 ng/ml, 7 days) and treated with PA for 24 h and then performed phagocytosis assays by incubating with *E. coli* bioparticles labeled with the fluorescent dye fluorescein. Results showed PA treatment significantly decreased phagocytosis in human macrophages (Fig. 6A, B). Next, we treated THP1-macrophages with PA for 24 h and labeled them with CytoTell blue, and performed efferocytosis assays using apoptotic T cells (Jurkat cells) fluorescently labeled with CFSE dye. The results showed that PA treatment significantly inhibited the efferocytosis of apoptotic Jurkat cells in THP1-macrophages (Fig. 6C, D). Overall, these functional assays matched our gene expression data by demonstrating that PA treatment significantly decreased phagocytosis and efferocytosis in human macrophages.

### Dysregulation of inflammation and efferocytosis genes in macrophages from a mouse model of diabetes plus accelerated atherosclerosis and human atherosclerosis, and after exposure to infectious agents

**Fig. 7:**
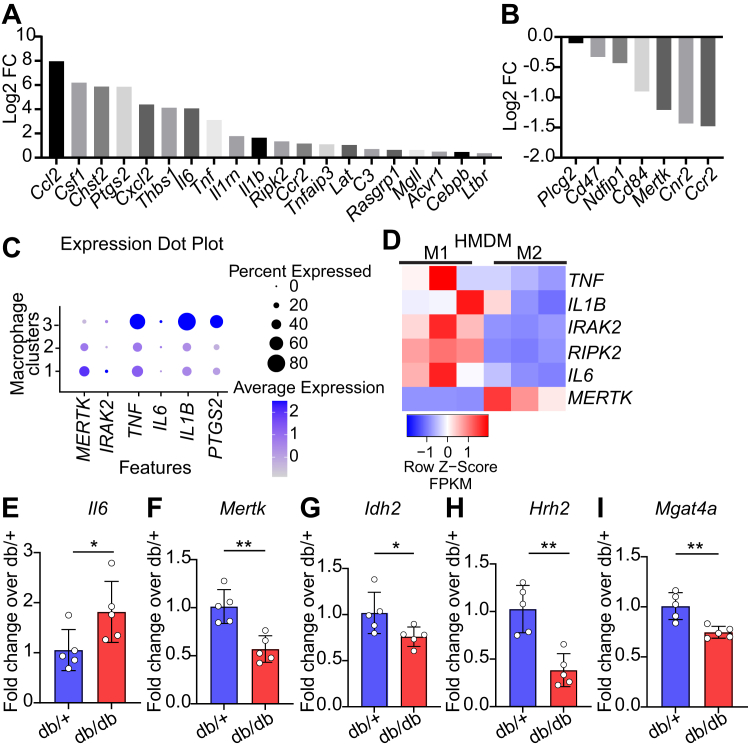
Inflammation-associated genes are upregulated and phagocytosis/efferocytosis genes are downregulated in macrophages in vivo in atherosclerosis and diabetes. A, B: Expression of PA-regulated enhancer-associated genes in bone marrow-derived macrophages from mice with accelerated atherosclerosis. Bar graphs show upregulated genes (A) and downregulated genes (B). C: Expression of inflammatory and efferocytosis genes in macrophage clusters 1–3 from human atherosclerotic plaques Data from GSE155512. were plotted using PlaqView, an open-source single-cell portal for cardiovascular research. D: Inflammatory response genes increased in human macrophages with the M1 phenotype, and *MERTK* expression downregulated in the M1 phenotype compared to the M2 phenotype. Heatmap showing the Gene expression analysis (FPKMs) from a published study GSE55536) of inflammatory genes *TNF*, *IL1B*, *IRAK2*, *RIPK2*, and *IL6* and efferocytosis gene *MERTK* in human macrophages with M1 and M2 phenotypes. E–I: qRT-PCR analysis of indicated genes in peritoneal macrophages from type 2 diabetic db/db or control db/+ mice. ∗*P* < 0.05 and ∗∗*P* < 0.01 as determined by unpaired *t* test for D–H (n = 5).

Next, we determined whether PA-induced dysregulated macrophage phenotypes are seen *in vivo* in macrophages from a mouse model of diabetes-induced accelerated atherosclerosis and chronic inflammation. We examined our earlier RNA-seq data from *Apoe*^−/−^ mice rendered diabetic with multiple injections of streptozotocin (model of diabetes-induced accelerated atherosclerosis) relative to non-diabetic *Apoe*^*−/−*^ mice. RNA-seq analysis was performed on bone marrow-derived macrophages (BMDM) isolated from diabetic *Apoe*^−/−^ mice (20 weeks of diabetes, when they depict increased atherosclerosis) versus non-diabetic *Apoe*^−/−^ controls (27, 30). Several genes associated with inflammation and immune-related processes were upregulated while genes related to phagocytosis and efferocytosis were downregulated in BMDM of the diabetic *Apoe*^−/−^ mice versus *Apoe*^−/−^ controls (Fig. 7A, B) similar to those in PA-treated human macrophages. Furthermore, we examined the publicly available single-cell RNA-seq data of macrophages from human subjects with atherosclerosis (41, 42) and evaluated gene expression in 3 different subsets of macrophage populations (based on clusters), Clusters 1, 2, and 3. Macrophage cluster 3 showed the highest levels of the inflammatory genes *TNF*, *IL6*, *IL1B*, and Prostaglandin-Endoperoxide Synthase 2 (*PTGS2*), which were much lower or not altered in macrophage clusters 1 and 2 (Fig. 7C). On the other hand, the expression of the efferocytosis-associated gene *MERTK* was lowest in macrophage cluster 3 and higher in macrophage cluster 1 and 2 (Fig. 7C). We also assessed gene expression in human macrophages polarized into classical M1 and alternatively activated M2 phenotypes (43) and observed increased expression of inflammatory cytokine genes (*TNF*, *IL1B*, *IRAK2*, *RIPK2* and *IL6*) in the M1 phenotype, while the expression of the efferocytosis-related gene *MERTK* was decreased in M1 phenotype compared to M2 human macrophages (Fig. 7D). Furthermore, we examined the expression of candidate PA-dysregulated genes in peritoneal macrophages isolated from db/db mice, a model of obesity, insulin resistance and T2D and chronic inflammation. RT-qPCR results showed that inflammatory genes like *Il6* were upregulated and efferocytosis associated gene *Mertk* was downregulated along with immune and metabolic genes like *Idh2, Hrh2* and *Mgat4*a in diabetic db/db mice versus control db/+ mice (Fig. 7E–I). Together these results showed that key genes regulated by PA in human macrophages that are associated with increased inflammation and decreased efferocytosis phenotypes were similarly observed in vivo in macrophages from human and mouse models of atherosclerosis and diabetes, supporting the relevance of our findings to cardiometabolic disease. We next analyzed the Cap Analysis of Gene Expression (CAGE) data available on FANTOM5 database to determine whether exposure to bacterial and fungal pathogens can regulate similar genes in human monocytes. We observed that bacterial and fungal pathogens significantly induced *IRAK2*, and *CSF2*, but significantly downregulated *MERTK* and *MGAT4A* genes (supplemental Fig. S2A–D). These results further suggest a link between chronic inflammation and defective phagocytosis and efferocytosis in macrophages during infection, further underscoring the key role of PA in metabolic diseases via dysregulation of monocyte/macrophage enhancers and functions related to innate immunity.

## Discussion

In this study, we report for the first time that PA treatment leads to extensive reprogramming of the enhancers/SE repertoire in human monocytes, which was associated with increased expression of inflammatory genes and reduced expression of phagocytosis and efferocytosis-related genes. The observed changes in gene expression correlated with PA-induced genomic interactions between enhancers and promoters of these genes. Furthermore, similar changes in gene expression were observed in macrophages from mice and humans with obesity, T2D, and atherosclerosis, pathologies known to be associated with elevated PA levels. Moreover, we observed that PA-regulated enhancers harbor genetic variations reported to be potentially involved in metabolic disease. These findings suggest that PA-induced epigenetic mechanisms at key genomic regulatory enhancers can contribute to monocyte and macrophage dysfunction associated with inflammatory cardiometabolic diseases.

We observed that PA increased several inflammatory genes like *IL1B* and *TNF*, which are closely associated with dysfunction of pancreatic islets, adipose, and skeletal muscle related to insulin resistance and hyperglycemia (44). PA-induced genes such as *RIPK2* and *IRAK2* are upstream regulators of NOD signaling and NF-κB activation involved in inflammatory gene expression (45, 46). We previously reported that PA induces *RIPK2* in human and mouse macrophages (30), but the mechanisms were not investigated. Here, we show that *RIPK2* and other inflammatory mediators like *IRAK2* are regulated by PA via putative enhancer-dependent mechanisms involving interactions between their promoters and cognate upstream enhancers/SEs. We observed several of these PA-regulated gene promoters show interactions with enhancers/SEs which were further increased after PA treatment. In contrast, genes associated with phagocytosis and efferocytosis, such as *HRH2* (47) and *MERTK*, and metabolism genes, such as *IDH2*, involved in M2 polarization (48), were downregulated, which could augment the inflammatory phenotype of macrophages. Downregulation of *MERTK*, a receptor tyrosine kinase, can impair efferocytosis and lead to inflammation and immune activation. We verified that PA suppressed efferocytosis of apoptotic T cells and inhibited phagocytosis of *E. coli* bioparticles, indicating functional outcomes for the downregulation of *MERTK* and *HRH2*. Furthermore, our data reveals a novel epigenetic mechanism for *MERTK* gene regulation by PA via dysregulated chromatin looping in human macrophages. It is possible that other downregulated genes shown in this study are regulated by similar mechanisms. These findings demonstrate the role of epigenetic mechanisms triggered by PA that regulate chromatin function at cis-regulatory elements.

Obesity, diabetes, and related cardiovascular complications are associated with increased inflammation, defective efferocytosis, and increased risk for infections. Our data clearly showed PA promotes upregulation of inflammatory genes and downregulation of protective genes involved in tissue homeostasis and immunity. These genes were similarly regulated in macrophages from diabetic *Apoe*^*--*^ mice with accelerated atherosclerosis in T2D db/db mice and in M1/M2 polarized human macrophages. Inactivation of the MERTK protein in atherosclerotic plaques via proteolytic cleavage impairs macrophage efferocytosis and promotes necrosis, defective resolution and progression of atherosclerosis (49). Thus, downregulation of *MERTK* by PA can also contribute to the defective efferocytosis and inflammation resolution associated with the pathogenesis of atherosclerosis. These data provide in vivo and disease relevance of our findings.

We observed that PA-altered enhancers overlapped with SNPs reported to be associated with diabetes and obesity, and these SNP-harboring enhancers were associated with genes altered in PA-treated human monocytes and macrophages. Dysregulated expression of such PA-upregulated genes e.g. *HDAC9*, *PCSK6*, and *CSF1* might contribute to the increased inflammation and risk for cardiovascular/cardiometabolic disease. *Hdac9* and *Apoe* double knockout mice depict decreased aortic lesion size compared to *Apoe* knockout alone. Moreover, deletion of *Hdac9* in bone marrow cells of *Ldlr*^*−/−*^ (low-density lipoprotein receptor knockout) mice decreased atherosclerosis. Furthermore, *Hdac9* deletion increased lipid homeostatic genes, and decreased inflammatory gene expression and polarized macrophages towards M2 phenotype (50). Interestingly, *Hdac9*^−/−^ mice were protected from diet-induced obesity and displayed improved insulin sensitivity and lower body weight (51). In macrophages and endothelial cells, HDAC9 was shown to activate IKKα/β and inflammatory response and was also implicated in atherosclerotic plaque instability (52, 53). It is possible that PA induced *HDAC9* is regulated by related enhancer SNPs to modulate macrophage functions associated with inflammation in metabolic disorders. Moreover, *PCSK6* upregulation plays an important role in macrophage activation in inflammatory and cardiovascular diseases (54, 55, 56). These results suggested that genetic variations in PA-regulated enhancers may affect the expression of these key genes to increase the risk for inflammatory metabolic diseases. However, clearly, more studies are needed to validate these observations in clinical cohorts and determine causality.

NF-κB TF regulates inflammatory gene expression by binding to promoters and enhancers in monocytes and macrophages (21). BRD4 protein can bind to H3K27ac at enhancers/SEs to modulate NF-kB activity and inflammatory gene expression (57). Notably, BRD4 inhibition blocks the communication between SEs and key target promoters, which is the mechanism of action of BRD4 inhibitors like JQ1 (40). Evidence shows myeloid-specific deletion of Brd4 in mice protected from inflammation and diet-induced obesity (58). Interestingly JQ1 suppressed atherogenesis in *Ldlr* ^*−/−*^ mice (24). Our results showed that JQ1 inhibited PA-induced inflammatory genes, supporting a role for BDR4-mediated enhancer coactivator functions in PA-induced gene regulation. Moreover, motifs for inflammation-associated TFs like NF-κB were enriched in PA-upregulated enhancers regions. We found that NF-κB signaling inhibitor BAY 11–7082 reduced PA-induced expression of inflammatory genes like *IRAK2* and increased the expression of *MERTK*, suggesting enhancer crosstalk between NF-κB and BRD4 in the regulation of PA-induced genes in human macrophages.

Taken together, our results identified epigenetic mechanisms regulated by PA at enhancers and SEs mediating the expression of genes involved in monocyte/macrophage dysfunction and showed the relevance of these changes to the pathology of cardiometabolic diseases. These dysregulated enhancers and associated genes represent novel therapeutic targets for inflammatory cardiometabolic diseases.

## Data availability

The RNA sequencing data were previously submitted to GEO under accession number GSE214160. The ChIP-seq data of H3K27ac are submitted to GEO under the accession number GSE281939.

## Supplemental data

This article contains supplemental data (33, 34).

## Conflicts of interest

The authors declare that they have no conflicts of interest with the contents of this article.
