# Supplementary material for: Palmitic acid alters enhancers/super-enhancers near inflammatory and efferocytosis-associated genes in human monocytes
Source: J Lipid Res. 2025 Mar 9;66(4):100774. doi: 10.1016/j.jlr.2025.100774 (PMC12002881; doi:10.1016/j.jlr.2025.100774)
Supplement: Supplemental Material [file mmc1.pdf]

Supplementary materials:

Palmitic acid alters enhancers/super-enhancers near inflammatory and efferocytosis-associated genes in human monocytes

Vinay Singh Tanwar[1], Marpadga A. Reddy[1], Suchismita Dey[1], Vajir Malek[1], Linda Lanting[1], Zhuo Chen[1], Rituparna Ganguly[1] and Rama Natarajan[1,2*]

Supplemental Figures

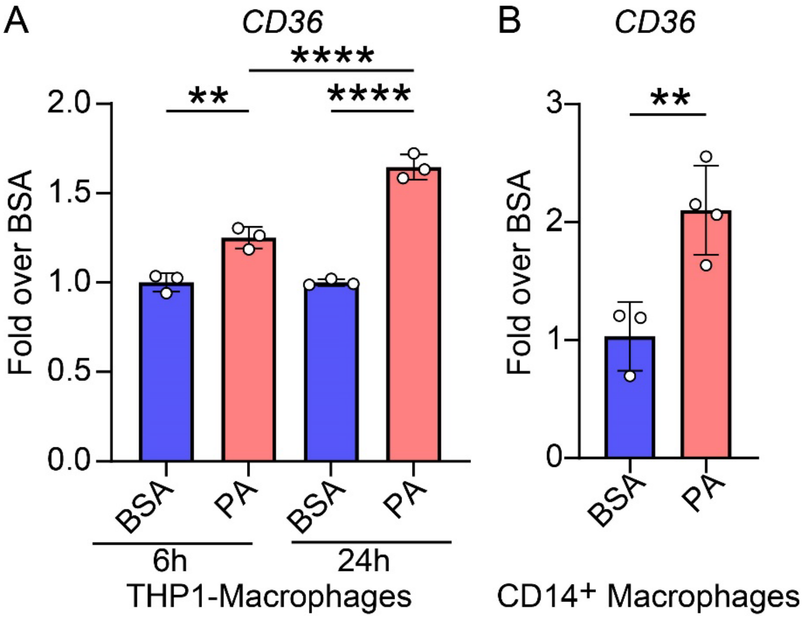

Supplemental Fig. S1: Palmitic acid upregulates the expression of *CD36* in human macrophages. A-B: Human THP1-macrophages (A) and CD14^{+}macrophages (B) were treated with palmitic acid (200µM) for 6 or 24h followed by RT-qPCR for determining *CD36* expression.

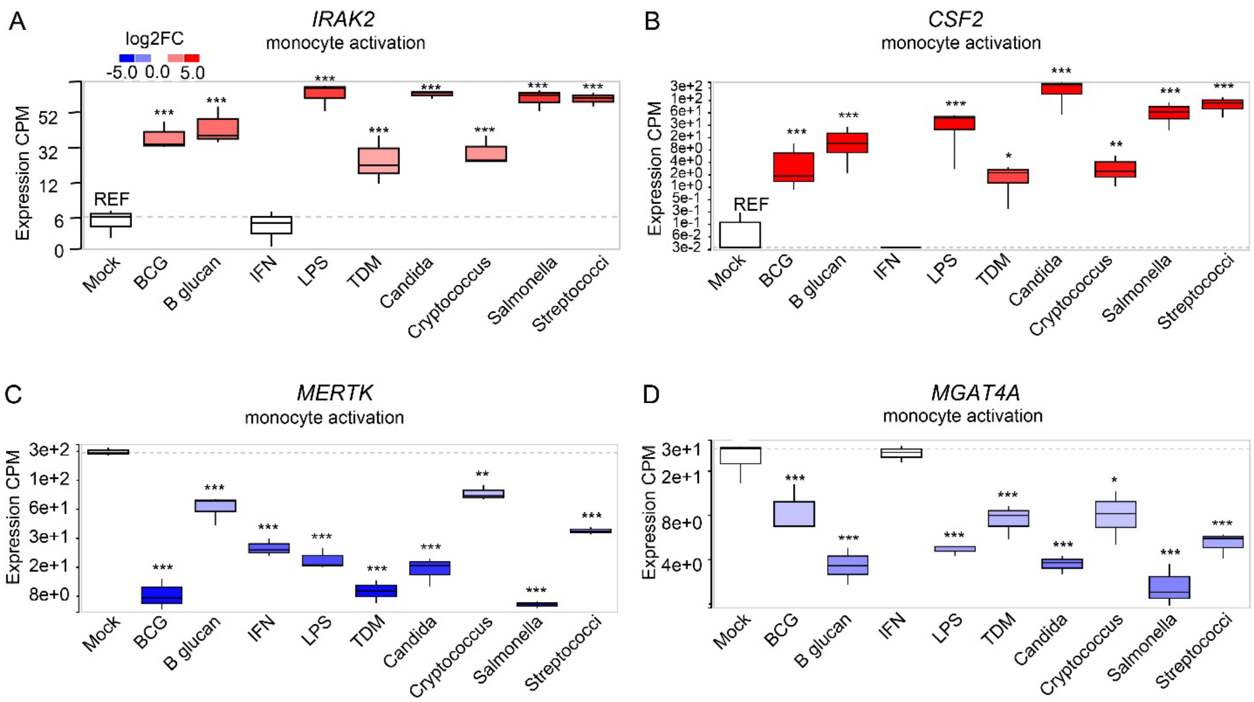

Supplemental Fig. S2: Inflammatory agents, bacterial and fungal pathogens alter expression of inflammatory and efferocytosis genes associated with PA-regulated enhancers/super enhancers.

A-D: Gene expression analysis of upregulated genes *IRAK2* (A) and *CSF2* (B) and downregulated genes *MERTK* (C) and *MGAT4A* (D) in human monocytes treated with Inflammatory agents, and bacterial and fungal pathogens. Graphs were downloaded from the FANTOM5 cap analysis of gene expression (CAGE) database (https://fantom.gsc.riken.jp/cat/v1/#/).

Supplemental Tables

Supplemental Table S1. Sequences of PCR primers. Human genes are indicated in uppercase letters and mouse genes are indicated in lowercase.

**Gene**	**Forward**	**Reverse**
RPLPO	GAAACTCTGCATTCTCGCTTC	GGTGTAATCCGTCTCCACAG
CSF1	TGGCGAGCAGGAGTATCAC	AGGTCTCCATCTGACTGTCAAT
GLIS3	GTTCAGCGACTGGGACTCATT	CCCTCTGTAAGCTAGGACTGAT
HDAC9	AGTAGAGAGGCATCGCAGAGA	GGAGTGTCTTTCGTTGCTGAT
PCSK6	CGCAGGCCCTTTACTTCAAC	CGGCAGCGACTGTTCTTGT
TPCN2	TCCCTGAGTCTCGTGTGTCAT	AGCATTCCGAACATGGTGAAG
AGRN	GTCCTGCGTCTGCAAGAAGAG	CTCGCATTCGTTGCTGTAGG
IL1B	CAAAGGCGGCCAGGATATAA	CTAGGGATTGAGTCCACATTCAG
TNF	CCCTGAAAACAACCCTCAGA	GTCCTTTCCAGGGGAGAGAG
RIPK2	ACCATTCCCTACCACAAACTC	AGTGTGGATGTGCAGGTG
OSBPL8	TCCTCATAGCCAGGGTTTTGA	AGGATCTGTGATTGTACTGAGCA
MERTK	AAATCCCCCTCCGTGCTAAC	TGGGGAGGGAATTGCTTTGA
IDH2	CAGTTCATCAAGGAGAAGCTCA	GTGGCCAGTGCAGAGTCAAT
HRH2	CGTGTCCTTGGCTATCACTGA	GGCTGGTGTAGATATTGCAGAAG
MGAT4A1	ACATGGTGTTGTAGCCAACCT	GCCCTTTTCTTGAGCATACATCA
CSF2	CTGAACCTGAGTAGAGACACTG	GCCCTTGAGCTTGGTGAG
IRAK2	GCTCACCCAATGGCTCATCT	CCTCGGCCAACACTATTCCA
CD36	GCCAGGTATTGCAGTTCTTTC	TGTCTGGGTTTTCAACTGGAG
Rplpo	TTATAACCCTGAAGTGCTCGAC	GCGCTTGTACCCATTGATG
Il6	GATGCTACCAAACTGGATATAATCAG	CTCTGAAGGACTCTGGCTTTG
Mertk	CAGGGCCTTTACCAGGGAGA	TGTGTGCTGGATGTGATCTTC
Idh2	GGAGAAGCCGGTAGTGGAGAT	GGTCTGGTCACGGTTTGGAA
Hrh2	CCCAATGGCACGGTTCATTC	GCCGACGATTCAAGCTGACA
Mgat4a	ATGAGGCTCCGAAATGGAACT	CCACTCGAAGACGCTCTTTTAG
**ChIP primers**		
CSF2	GGCTTTCTACCTAAGAACCCTG	GCAGTTTCCCATTGTCCTTG
RIPK2	AGGTGTGGGAATAAAGAGCAG	AACCGTGAATGTCCTTGGG
IRAK2	TGTCACAGCCCTTTCTTGTAG	CATCCCTCCAAACCTGCTC
MERTK	ACTTGACTTTCCTTGAGCCC	GCCACAAATTGAGAACAGCC
**3C Primers**		
IRAK2 E3 P1	GAGTGGTAACTGAGATGTGTCC	GGCAGAGACAGGAAGTTGATT
IRAK2 E3 P2	CTTCTGTTTCTAGGAAATTCAATTAGAAGG	AGACAGGAAGTTGATTGTCTATCTTTG
IRAK2 E3 P3	CTTCTGTTTCTAGGAAATTCAATTAGAAGG	TTCTCTACCCCTTGAGACCA

IRAK2 E3 P4	CTTCTGTTTCTAGGAAATTCAATTAGAAGG	TACGTACATGTGTTTTAAAGAGAGAAGTTAT
MERTK E3	CCCAGCTGGAAGAATGAAAAGTT	GGTTGTACATTTCTGAGTC
MERTK E4	CCCAGCTGGAAGAATGAAAAGTT	TATATAGGAGGCACAGTG

Supplemental Table S2. Summary of enhancers classified based on the impact of PA and overlap with SNPs associated with T2D, BMI and obesity.

SNP analysis in PA-regulated enhancers: The SNPs associated with T2D, BMI and obesity (7830) were obtained from published GWAS studies (1). PA-regulated enhancer regions (FDR<0.05, n=861) and non-PA-regulated enhancers (FDR>0.05, N=9370) overlapping with these SNPs were identified using intersectBed command in bedtools (2). Two-sided Fisher's exact test was used to determine the statistical significance of enrichment of these SNPs in PA-regulated enhancer regions (*odds ratio=2.38, p-value = 0.0001).

		Total Enhancers	Enhancers Classified based on SNPs	
			Contain SNPs of interest	Not Contain SNPs of interest
Classified based on PA effect	PA-regulated	861	30*	831
	Not PA-regulated	9370	140	9230

Supplemental Table S3. PA-regulated enhancers overlapping with SNPs associated with obesity, Body mass index (BMI), and type 2 diabetes (T2D).
Table showing the list of PA-regulated enhancers (enhancer location and H3K27ac enrichment (log2FC)) containing SNPs associated with obesity, Body mass index (BMI), and type 2 diabetes (T2D). The reported values for SNPs (reported genes, mapped gene, disease/trait and genetic association p-values) are from GWAS catalog. NR = not reported; NA= not applicable.

S. No	Enhancers Location	Enhancer (log2FC)	SNP	REPORTED GENE(S)	MAPPED GENE	DISEASE / TRAIT	Genetic Association P-VALUE
1	chr1:1004335-1006342	3.22	rs3934834	NR	AGRN - RNF223	BMI	6E-07
2	chr4:1290328-1300797	-1.13	rs13108904	MAEA, KIAA1530	MAEA	Obesity-related traits	0.000002
3	chr16:4009383-4015619	1.44	rs2531995	ADCY9	ADCY9	BMI	0.000001
			rs2238435		ADCY9	BMI	2E-31
4	chr19:18474357-18492051	0.95	rs10424912	NR	GDF15	BMI	6E-11
5	chr20:23091838-23143355	0.70	rs1320561	LOC200261, CD93	CD93 - LINC00656	Obesity-related traits	0.000001
6	chr8:23385265-23413552	0.76	rs11779446	NR	SLC25A37	BMI	8E-10
			rs11781222	NA	SLC25A37	BMI	3E-12
7	chr20:30637121-30681596	-1.01	rs1987960	NR	HCK	BMI	5E-09
8	chr16:31358145-31371716	0.62	rs7190997	NR	ITGAX	BMI	1E-13
9	chr15:45926143-45950357	0.81	rs607541	SQRDL	SQOR	Obesity-related traits	0.000008
10	chr17:46122604-46135943	0.98	rs3764400	CBX1	NFE2L1-DT	BMI	4E-07
11	chr1:54694649-54697810	2.42	rs4061073	SSBP3	SSBP3	BMI	0.000004
12	chr15:63766106-63798839	0.53	rs56187480		LINC02568 - USP3	BMI	3E-09
			rs144268704		LINC02568 - USP3	BMI	1E-09
13	chr11:72443929-72453561	0.74	rs7123876	NA	ARAP1	BMI	3E-10
14	chr16:79360480-79365554	-2.89	rs7191820	intergenic	RNA5SP431 - NA	Obesity-related traits	0.000006
15	chr1:110438106-110445581	2.55	rs333960	CSF1	LINC01768 - CSF1	Obesity-related traits	2E-07
16	chr6:113948836-113978051	-0.50	rs9384878		LINC02541 - MARCKS	BMI	2E-08
17	chr2:113991466-114002435	1.11	rs13405033	NA	PAX8-AS1, PAX8	BMI	2E-07

			rs58603829	IL10RA	TMPRSS13 - IL10RA	Obesity-related traits	0.000006
18	chr8:132861568-132872821	1.05	rs11997238	NR	NA - EFR3A	BMI	6E-09
19	chr6:139639614-139667755	-0.59	rs11155053	CITED2	TXLNB - CITED2	Obesity-related traits	0.000009
20	chr5:169712608-169731181	0.72	rs3804250	LCP2	LCP2	BMI and Diastolic blood pressure (bivariate analysis)	0.000005
21	chr1:150971394-150975892	2.35	rs140386498	FAM63A	MINDY1	T2D	8E-08
22	chr11:68845739-68849177	1.51	rs35264875	TPCN2	TPCN2	T2D	0.000002
23	chr12:111834356-111838378	1.19	rs149212747	SH2B3, ALDH2	LINC02356	T2D	2E-11
24	chr14:103881897-103895654	0.88	rs62007683	MARK3	MARK3	T2D	3E-08
25	chr15:101984844-101997560	2.23	rs6598475	PCSK6	PCSK6	T2D	2E-08
26	chr17:46122604-46135943	0.98	rs9900074	NR	NFE2L1-DT	T2D	3E-10
27	chr2:145258958-145283355	-0.57	rs2033159	NR	ZEB2	T2D	5E-10
28	chr4:164527290-164536682	-1.31	rs3792615		MARCHF1	T2D	0.000009
29	chr7:18542661-18550451	1.41	rs11298745	HDAC9, MIR1302-6, TWIST1	HDAC9	T2D (age of onset)	0.000002
30	chr9:4291997-4302321	1.29	rs4258054	GLIS3	GLIS3	T2D	6E-08
			rs10814916	GLIS3	GLIS3	T2D	6E-12
			rs10758593	GLIS3	GLIS3	T2D	3E-07

References

1. Sollis, E., A. Mosaku, A. Abid, A. Buniello, M. Cerezo, L. Gil, T. Groza, O. Gunes, P. Hall, J. Hayhurst, A. Ibrahim, Y. Ji, S. John, E. Lewis, J. A. L. MacArthur, A. McMahon, D. Osumi-Sutherland, K. Panoutsopoulou, Z. Pendlington, S. Ramachandran, R. Stefancsik, J. Stewart, P. Whetzel, R. Wilson, L. Hindorff, F. Cunningham, S. A. Lambert, M. Inouye, H. Parkinson, and L. W. Harris. 2023. The NHGRI-EBI GWAS Catalog: knowledgebase and deposition resource. *Nucleic Acids Res* **51**: D977-D985.

2. Quinlan, A. R., and I. M. Hall. 2010. BEDTools: a flexible suite of utilities for comparing genomic features. *Bioinformatics* **26**: 841-842.
